# In silico designing of a recombinant multi-epitope antigen for leprosy diagnosis

**DOI:** 10.1186/s43141-022-00411-7

**Published:** 2022-09-02

**Authors:** Marcela Rezende Lemes, Thaís Cristina Vilela Rodrigues, Arun Kumar Jaiswal, Sandeep Tiwari, Helioswilton Sales-Campos, Leonardo Eurípedes Andrade-Silva, Carlo Jose Freire Oliveira, Vasco Azevedo, Virmondes Rodrigues, Siomar C. Soares, Marcos Vinicius da Silva

**Affiliations:** 1 Department of Immunology, Microbiology and Parasitology, Institute of Biological and Natural Sciences, Federal University of Triângulo Mineiro (UFTM), Uberaba, Minas Gerais 38025-180 Brazil; 2 Laboratory of Cellular and Molecular Genetics (LGCM) Department of Genetics, Ecology, and Evolution, Institute of Biological Sciences,, Federal University of Minas Gerais (UFMG), MG 31270-901 Belo Horizonte, Brazil; 3 Institute of Tropical Pathology and Public Health, Federal University of Goiás (UFG), Goiânia, Goiás Brazil; 4 Infectious Disease Department, Institute of Health Sciences, Federal University of Triângulo Mineiro (UFTM), Uberaba, Minas Gerais Brazil

**Keywords:** Chimeric protein, Diagnosis, Hansen’s disease, Immunoinformatics, Leprosy

## Abstract

**Background:**

Leprosy is caused by *Mycobacterium leprae* and *Mycobacterium lepromatosis*. Most of the affected population lives in low-income countries and may take up to 10 years to show any clinical signs, which is how physicians diagnose it. However, due to progressive cell damage, early diagnosis is very important. The best way to confirm leprosy is through bacilloscopic, which only confirms the diagnosis and has low accuracy or PCR, that requires specialized operators and is expensive. Since the bacteria are fastidious and do not grow in any culture media, therefore, diagnosing leprosy in the lab is still a challenge. In this concern, a recombinant multi-epitope protein can be a beneficial strategy in the management of the diagnosis, as diverse immunogenic epitopes are precisely selected to detect specific antibodies. Therefore, the purposes of the present study were to select immunogenic epitopes from different relevant proteins, with immunogenic properties, and then to construct a recombinant multi-epitope protein that accuses the presence of the antibodies in the early stages of the disease, making it more than appropriate to be applied as a diagnostic tool.

**Results:**

We selected 22 common proteins from both species and, using bioinformatics tools, predicted B and T cell epitopes. After multiple filtering and analyzing, we ended up with 29 epitopes {MHC-I (total 18) and MHC-II (total 11)} from 10 proteins, which were then merged into one construct. Its secondary and tertiary structures were also predicted and refined to comprise the amino acid residues in the best conformation possible. The multi-epitope protein construct was stable, non-host homologous, non-allergic, non-toxic, and elicit humoral and cellular responses. It has conformational B cell epitopes and potential to elicit IFN-γ, IL-4, and IL-10 secretion.

**Conclusions:**

This novel recombinant multi-epitope protein constructed using the common epitopes from *M. leprae* and *M*. *lepromatosis* has a huge immunological potential, is stable, and can be lyophilized to be used in ELISA plates or even in biosensors, which are user-friendly diagnosis tools, facilitating translation into human sample tests.

**Supplementary Information:**

The online version contains supplementary material available at 10.1186/s43141-022-00411-7.

## Background

Only in 2019 the WHO (World Health Organization) reported 202,226 new cases of Leprosy worldwide, with almost 80% of the cases in just 3 countries: India, Brazil, and Indonesia (114,451, 27,863, and 17,439, respectively) [1, 2]. Leprosy is caused by *Mycobacterium leprae* and *Mycobacterium lepromatosis*, which can invade Schwann cells [3] affecting both the dermis and peripheral nerves [4]. The cell invasion causes nerve demyelination through nerve cell communication deregulation [5]. The damage to the myelin causes permanent loss of thermal and tactile sensibility, besides pain sensation [4]. Leprosy may take up to 11 years until any clinical manifestation occurs, but even before that, it is transmissible [6, 7].

The immunological response in leprosy is highly dependent on the host’s genetic background, and it drives its clinical manifestations [6, 7]. The leprosy spectrum ranges from tuberculoid leprosy (TL) to lepromatous leprosy (LL). In TL, the immune response has mainly a cellular profile, with a Th1 response, producing cytokines like interferon gamma (IFN-γ), interleukin (IL)-2, and IL-12. On the other hand, the LL pole has a Th2/Th17 response, with more antibody titers; IL-10, IL-4, and IL-13 secretion;, and a higher bacillary load [8, 9]. Between those poles exist borderline tuberculoid (BT), borderline-borderline (BB), and borderline lepromatous (BL) clinical manifestations, with mixed immunologic characteristics, ranging from the Th1 profile to the Th2/Th17 according to the poles [10]. The borderline presentation is the one that most of the patients fit, and the nerve involvement is more severe, causing higher levels of disability [11].

The leprosy diagnosis is mainly based on clinical and laboratorial evaluations. Due to the progressive cell damage, the early diagnosis is very important; however, most diagnoses are performed when there is already a significant nerve damage [12]. The best way to confirm leprosy is through PCR, which requires specialized operators, is expensive, and is very difficult to be conducted in the field. Another option is bacilloscopic, which is not used as a diagnosis, only as confirmation of the clinical diagnosis, and has low accuracy. Serological tests exist only based on *M. leprae* and are not sensitive enough, detecting only LL and symptomatic cases, but not PB [13–15]. The bacteria are fastidious and do not grow in any culture media; therefore, diagnosing leprosy in the lab is still a challenge [8]. Given the different immunological responses, van Hooij et al. established that the combination of humoral and cellular detection is efficient in diagnosing both MB and PB [16, 17].

To try and stop the transmission of new leprosy cases, the WHO set a few objectives to be fulfilled from 2016 to 2020. One of the objectives was the development of a new diagnosis tool [18]. Since most of the affected population lives in lower-income countries, they do not have access or cannot afford the number of tests necessary for all the population at risk [16, 19], and the serum or whole blood-based assays are not conclusive for all types of leprosy [16]. This situation leaves the population with only the possibility of discovering the disease after clinical manifestations, increasing the transmission [20, 21] and making it difficult to finish the cycle.

Bioinformatics tools are of the utmost utility to assess the immunogenic peptides within a protein, saving time, money, and even diminishing the use of animals, since it provides multiple filters before in vitro and in vivo tests are performed [22–25]. Given that *M. leprae* and *M. lepromatosis* are not yet cultivable in any culture media [8], bioinformatics is the best way to assess its proteins and their immunogenic potential. It also provides the possibility to create recombinant multi-epitope constructs, which can hold several antigenic epitopes, differing from natural proteins or whole-cell preparations [26]. Multi-epitope proteins can be used in enzyme-linked immunosorbent assay (ELISA), lateral flow tests, biosensors, and cellular assays, which require minimal or zero sample preparation. These constructs may also increase the sensitivity and specificity of the detection method, given that it is possible to assess homology relations with other microorganisms and permits the fusion of epitopes from different proteins and different sites of the organism. The multi-epitope constructs enable a higher immunogenic density and diminish the cross-reaction risk of whole bacteria antigen, which is common in leprosy diagnosis [27]. Diseases such as hepatitis B [23], Chagas disease [28], cryptococcosis [25], leishmaniosis [29, 30], tuberculosis [31], and toxocariasis [32] already have great results with recombinant multi-epitope proteins in their diagnosis.

The largest challenge of the multi-epitope diagnosis is to construct a chimeric protein with all the epitopes exposed to interact with the antibodies, as the coiling event predicted can be different in practice [33]. Furthermore, despite the advances in genetic engineering and recombinant expression technologies, some obstacles endure in protein production as toxicity, instability, inability to fit the environment, and errors in expression vector selection, among others [34].

There is an effective therapy against leprosy, the multidrug therapy (MDT) program [35]; however, if the diagnosis is not made early in the disease, the nerve damage is unrecoverable, causing persistent physical damage [36]. Hence, to improve the diagnostic tools for this severe disease, we propose a novel recombinant multi-epitope-based antigen, using bioinformatics tools. To be able to diagnose both strains that cause the disease, 22 common proteins of *M. leprae* and the recently described *M*. *lepromatosis* were selected.

The purposes of the present study were to select deeply immunogenic epitope proteins, with immunogenic properties or certified to be detected in diagnosis tests, and then to construct a recombinant multi-epitope protein that could be applied as a diagnostic tool. Advanced techniques in protein structure design and evaluation were performed to build a stable and safe multi-epitope protein. Also, in silico cloning was applied to arrange codon bias to get an idea about the capacity of the protein to be expressed.

## Methods

**Fig. 1:**
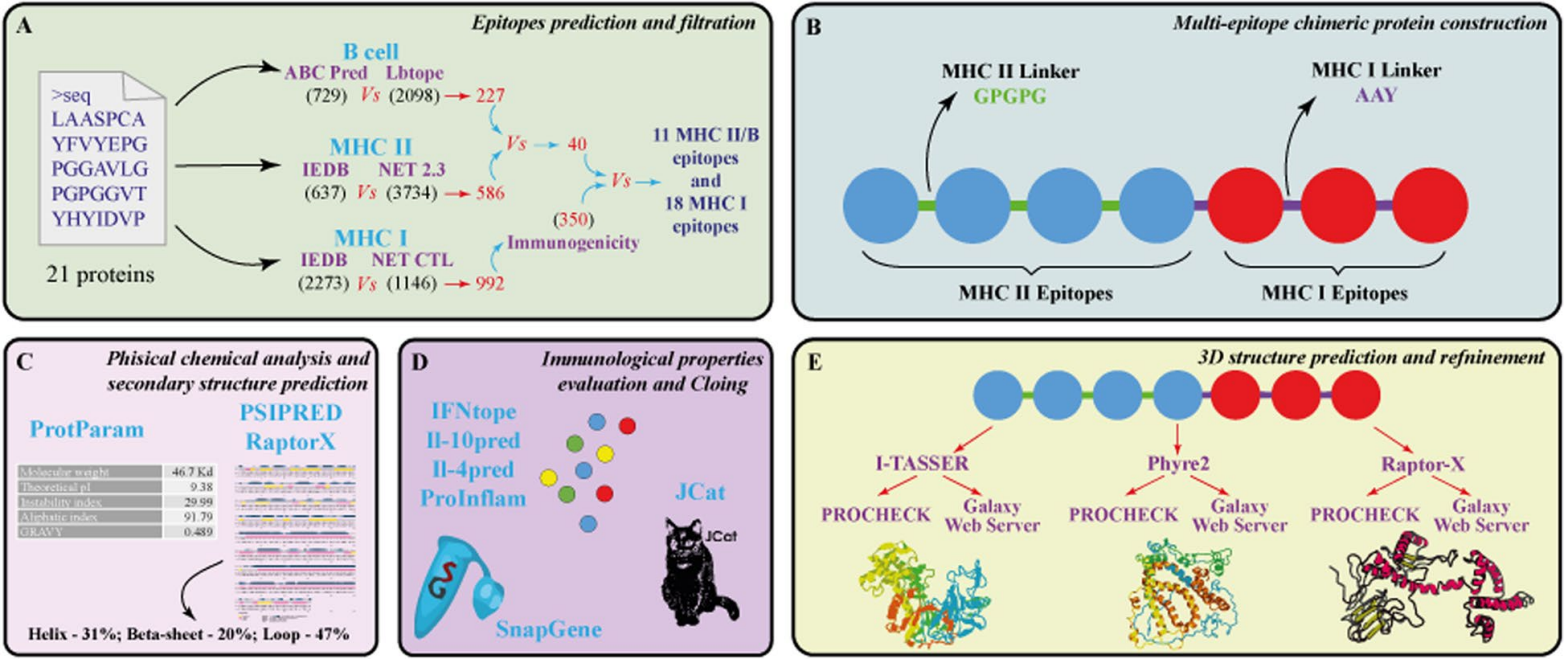
**A**–**E** Graphical representation of the pipeline used in this study to design a universal recombinant multi-epitope antigen for leprosy diagnosis

The complete workflow of the methodology used in this study is described in Fig. 1.

### Data selection

The *M. lepromatosis* and *M. leprae* proteins ML0091, ML0405, ML1636, ML2055, ML2331, ML2346, and ML1556 were previously proved to detect leprosy at some level [26]. ML2028, ML2055, ML2380, and ML2531 were tested as immunizers in mice, and they demonstrated reduced bacterial burden [4]. NP_301196.1, NP_301663.1, NP_301805.1, NP_301958.1, NP_302056.1, NP_302185.1, NP_302232.1, NP_302292.1, NP_302342.1, NP_302490.1, and NP_302503.1 were obtained as immunogenic proteins from our previous results, through reverse vaccinology analysis [37]. The sequences of these proteins were retrieved from National Center for Biotechnology Information (NCBI) in FASTA format [38]. The antigenicity of these selected proteins was evaluated by VaxiJen [39]. In total, 22 proteins shared among both strains were used for the next steps.

### Prediction of epitopes that binds to MHC I alleles

The epitopes able to bind to MHC I alleles and activate cytotoxic T lymphocytes (CTL) were predicted by two different platforms to improve the confidence of the prediction. The Immune Epitope Database and Analysis Resource (IEDB) contain thousands of high- and low-affinity epitopes used in training to enhance the accuracy of the predictor [40, 41]. Aiming to develop a diagnostic tool to be used in all endemic areas, we selected all 27 alleles with high frequency in the global population. The lengths of our peptides were 9 amino acid residues [42]. Default parameters were chosen for the prediction since they combine artificial neural network (ANN), scoring matrix method (SMM), and combinatorial library. Epitopes with percentile rank smaller than 1% were selected for our study, due to their enhanced probability to be immunogenic. NetCTL-1.2 server can assess binding affinity, antigenic processing, and transportation, integrated into the epitope prediction, using both ANN and SMM to make the predictions [43, 44]. The same alleles used in IEDB were used in NetCTL-1.2.

### Prediction of epitopes that binds to MHC II alleles

For epitopes that activate helper T lymphocyte (HTL) (MHC II-binding epitopes), we also used two different predictors, IEDB tool [40] and NetMHCII-2.3 server [45]. The MHC II cleft size can accommodate epitopes from 13 to 25 amino acids; thus, we chose to use a 15-residue length as a standard, since the NetMHCII-2.3 server allows users to use this length, approving the comparison between both programs. In IEDB, we selected only epitopes with percentile rank lower than 3%. For the IC50, which is used to determine the epitopes’ affinity with the MHC, we chose an IC50 < 1000 nM [45]. ANN is also used by the NetMHCII-2.3 server with various epitope databases to increase data training and predict the epitopes [46].

### Prediction of B cell epitopes

To predict linear B cell epitopes, we used ABCpred [47, 48] which uses ANN for predictions and LBtope server [49] which uses the support vector machine (SVM)-based models for the prediction. We chose the epitope’s length as 16 due to its better accuracy properties [48, 50, 51].

### Filtering and immunogenicity assessment of MHC I epitopes

All the epitopes predicted were filtered through an *in-house python* script which compare the results from both programs for each epitope (Fig. 1A). After the recognition of epitopes predicted by the two programs, the same script was used to find overlapping epitopes between B cells and MHC II with at least nine sequential amino acid residues. The last time that the script was used was to search for the overlap between class I epitopes predicted as immunogenic by the immunogenicity tool and the remaining class I epitopes. Class I immunogenicity tool [52] uses amino acid properties and their position within the peptide to predict immunogenic properties. Only peptides with a score greater than 0.1 were chosen.

### Sequence construction

The epitopes that passed through all those filters were then merged into different constructs with the sequence AAY for MHC I epitopes and GPGPG for MHC II as peptide linker sequences, which help in protein folding [53].

### Evaluation of host homology and physical–chemical properties

To evaluate the similarity between the constructed protein with human proteins, and therefore reduce autoimmunity possibilities, a BLASTp was carried out. The whole multi-epitope protein sequence and its individual epitopes were submitted against the UniProtKB Human database.

Molecular mass, theoretical pI, extinction coefficient, aliphatic index, grand average of hydropathicity (GRAVY), estimated half-life for three model organisms (*Escherichia coli*, yeast, and mammal cells), and the instability index were analyzed through the final construct sequence using ProtParam [54]. Solubility index was also assessed by Protein-Sol [55], which evaluates several properties based on *E. coli* expression data.

### Secondary structure prediction

The secondary structure of the final epitope construct was predicted by RaptorX template-based protein structure modeling server [56] and PSIPRED. PSIPRED predicts the secondary structure and generates the pictures by applying complex ANN and position-specific scoring matrix (PSSM) [57].

### Structural modeling, refinement, and properties assessment

To predict the tertiary structure (3D), three different programs were used, and the best 3D structure was chosen based on its structural quality. For the evaluation, PROCHECK was used through SAVES v6.0 [58, 59] to generate the Ramachandran plot. Phyre2 intensive method comprises the multiple alignments of the sequence of interest with homologous sequences using threading and ab initio techniques followed by the secondary structure’s prediction with the PSIPRED. Then, a hidden Markov model (HMM) is determined with the information from these two steps combined. The models with the best scores are used, from a search in an HMM database of known protein structures, to determine the modeling and error correction [60]. Multiple-template threading (MTT) and scoring methods are used in RaptorX to predict the 3D structures and to indicate the quality of models predicted [56]. Finally, I-TASSER uses an interactive method based on the templates according to fragment assembly simulations with further refinement to construct the models [57].

To enhance he local and global quality of the modeled 3D structure, we used GalaxyWeb Server which applies the methods for the refinement of amino acid side chains using light and aggressive relaxation approaches [61].

### Antigenicity, IFN-γ, IL-4, and Il-10 inducing potential

The final construct sequence was analyzed for crucial aspects related to the induction of immune responses, toxicity, and allergenicity. We used VaxiJen to assess the antigenic capacity through the automatic cross-covariance method, thus analyzing the physical–chemical properties and predicting the ability to induce immune responses without the need to do alignments [39].

The search for epitopes able to induce IFN-γ production was performed with the IFNepitope predictor, using MHC II epitopes. This predictor uses a SVM hybrid method based on motifs to perform the prediction [62]. IL-4 and IL-10 inductions were also assessed by different predictors (IL-4Pred and IL-10Pred), by the same method [63, 64]. The ProInflam web server was used as well to predict the pro-inflammatory potential of the peptides included in the protein [65].

### Conformational B cell epitopes prediction

The ElliPro web-based tool was used to predict conformational B cell epitopes from the refined predicted structure of our multi-epitope protein [66]. These epitopes are generally conformational, which means they are away in linear distance but close in spatial proximity [67].

### In silico cloning

To verify the capacity of cloning and expression of the multi-epitope protein in an appropriate expression vector, we performed in silico cloning. Using JCat, we adapted the codon of our peptide according to the *E. coli* K12 expression system’s codon usage through reverse translation. With the cDNA-optimized sequence, the codon optimization for *E. coli* k12 was performed, and it returned the Codon Adaptation Index (CAI), which must have a score higher than 0.8, and the GC content rate should be between 30 and 70%. Furthermore, to clone the final optimized gene sequence, we used the pET28a( +) vector obtained from the Addgene website (https://www.addgene.org/), with *Blpi* and *BamHI* restriction sites. Finally, the optimized sequence was inserted into the pET28a( +) vector using the SnapGene tool [68] to ensure protein expression.

## Results

### Prediction of B, CTL, and HTL epitopes

All the selected proteins had predicted antigenicity, assessed by VaxiJen analysis, showing their capacity to recognize peptides of immunological relevance (Additional file 1: Table S1). The 21 proteins submitted at ABCpred generated a total of 729 epitopes and 2098 in LBtope. Using the *in-house* python script, we searched for overlapping epitopes that were predicted by both programs, in order to find common epitopes, and it returned 227 B cell shared epitopes (Additional file 1: Table S2). For cytotoxic T lymphocyte (MHC I), we used IEDB MHC-I binding predictions and NetCTL 1.2 server. The first program predicted 2273 epitopes and the latter 1146, with 992 common epitopes predicted by the two software (Additional file 1: Table S3). IEDB MHC-II binding predictions and NetMHCII 2.3 were the tools used for MHC II epitope prediction, with 637 and 3734 epitopes, respectively. There were 586 common epitopes found (Additional file 1: Table S4).

### Epitope’s screening

To find epitopes with the potential to induce both humoral and cellular immune responses, we applied the *in-house* python script, searching for overlaps between MHC II (637) and B (227) epitopes, with similarity of at least nine sequential residues. At this screening step, we reduced the total number of MHC II and B epitopes to 40 overlapping epitopes (Additional file 1: Table S5). By applying the class I immunogenicity tool, we predicted the 350 most immunogenic epitopes, with scores greater than 0.1 (Additional file 1: Tables S5 and S6), using the previous common MHC I epitopes (992). Those 350 epitopes were, then, overlapped with the 40 ones resulting from humoral and cellular overlap (MHC I with B cell epitopes overlapping), giving a total of 20 epitopes (Additional file 1: Table S5) Fig. 1A.

From the 20 selected MHC I epitopes, we compared the sequence and its percentile rank. MHC I epitopes with only one or two residues of difference were excluded, using the lowest percentile as a parameter. As a result, a total of 29 {MHC-I (total 18) and MHC-II (total 11)} epitopes, from 10 proteins, were selected for the final recombinant multi-epitope construction (Table 1).

**Table 1:** Final list of the selected epitopes

Protein	B/MHC II	Percentile rank	MHC I	Percentile rank
ML0091	TLAIASPCAYFLVYEP	8.3	SPCAYFLVY	0.05
ML2346	AVLWELGYRRFAYVDQ	5.5	LGYRRFAYV	0.95
			VLWELGYRR	0.35
			ELGYRRFAY	0.13
	GVTYHYIDVPARTFAS	3.7	YIDVPARTF	0.23
ML2380	HWGNWAKIFFNNKGVV	6.2	HWGNWAKIF	0.31
NP_301196.1	RWKWHDPYVHASLLAQ	2.6	RWKWHDPYV	0.53
NP_301958.1	GVLIFAAILVTGFLWP	2.7	VLIFAAILV	0.18
			VTGFLWPAW	0.88
			LVTGFLWPA	0.16
			FLWPAWLVT	0.12
			FAAILVTGF	0.52
			AILVTGFLW	0.51
NP_302056.1	MSTIFGQVTTKEKQCQ	1.4	IMSTIFGQV	0.16
NP_302185.1	VLVFDAHRGMVVGSPL	8.1	LVFDAHRGM	0.25
NP_302292.1	TNIGLVSCKRDVGAAV	2.4	MVVTNIGLV	0.25
NP_302342.1	TRFVAAHGAYLVWLEQ	1.1	FVAAHGAYL	0.1
NP_302503.1	TFTKPEILTRYLNLVS	2.1	KPEILTRYL	0.06

### Multi-epitope sequence construction: structural modeling, refinement, and properties assessment

Different amino acid sequences were constructed with the selected epitopes to evaluate and select the one with the best structure quality. To give our construct flexibility to make its conformational changes, we joined these sequences with different peptide linkers since they assist in protein folding, which is important for the conformational epitopes [67]. For MHC I, we used AAY linkers, and for MHC II, we used GPGPG, forming a 431-amino acid multi-epitope protein; however, we made changes in the positions of epitopes. All sequences were submitted to the structural prediction in I-TASSER, Phyre2, and RaptorX. Afterward, a Ramachandran plot was constructed for all the amino acid sequences.

**Fig. 2:**
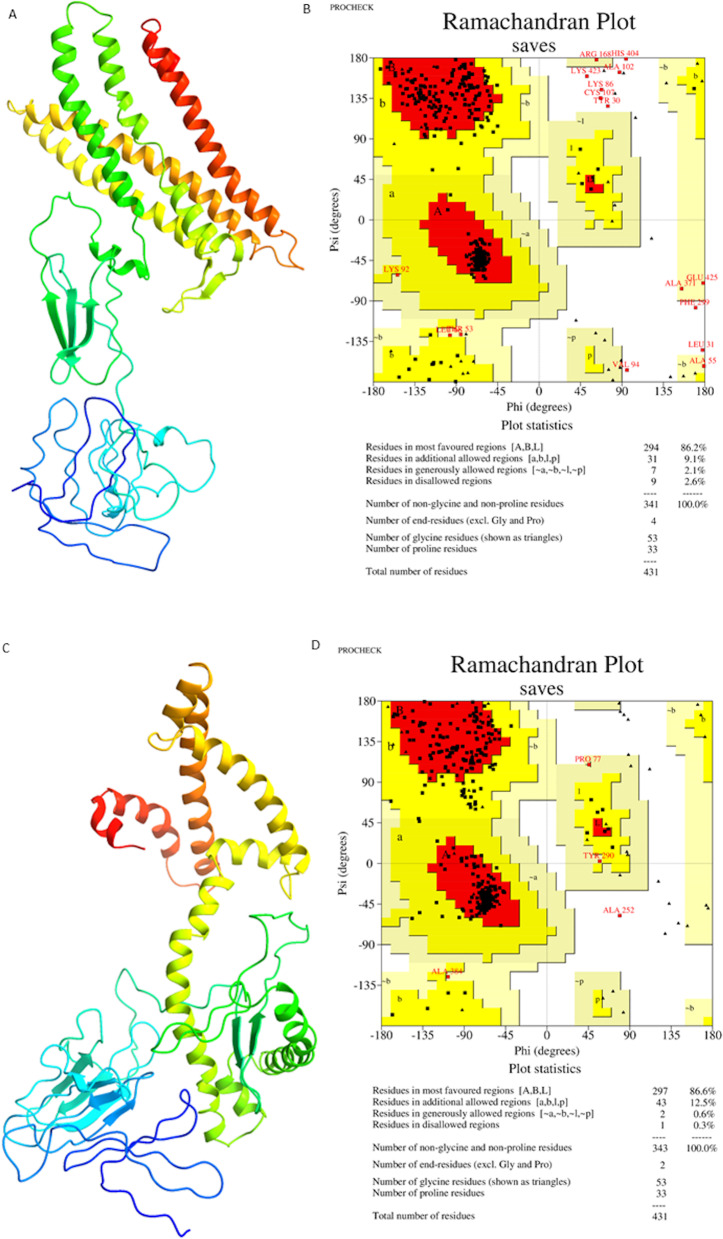
3D structure and the Ramachandran plot of the recombinant protein structure before and after. **A** Tertiary structure generated by the Phyre2 server. **B** Ramachandran plot for 3D structure generated by Phyre2 showed 86.2% of the residues in favored regions, 9.1% in allowed regions, and 2.6% in disallowed regions. **C** Representing the refined tertiary structure obtained by the GalaxyRefine server. **D** Ramachandran plot for the 3D structure generated by GalaxyRefine showed 86.6%, 12.5%, and 0.3% of the residues in favored, allowed, and disallowed regions, respectively

The best structure quality obtained was the one modeled by RaptorX, with 86.2% of the residues in the most favored regions, 9.1% in the additional allowed regions, 2.1% in the generously allowed regions, and 2.6% in the disallowed regions (Fig. 2A, B). We performed the refinement with GalaxyWeb Server, getting the best result with model 5, with 86.6% of the residues in the most favored regions, 12.5% in the additional allowed regions, 0.6% in the generously allowed regions, and 0.6% in the disallowed regions (Fig. 2C, D).

### Secondary structure prediction

According to PSIPRED and RaptorX, the predicted secondary structure has 431-residue protein, 31% helix, 20% beta-sheet, and 47% loop formation (Additional file 1: Fig. S1).

### Host homology and physical–chemical properties

Host homology was performed through NCBI BlastP with *Homo sapiens* (taxid 9606), and no significant similarity was found.

The protein’s molecular mass is 46,779.35 (46.7 kDa), with a theoretical pI of 9.38, which means its behavior is at basic pH. The protein is considered stable since the instability index is 29.99. The aliphatic index is 91.79, also showing stability in changes in temperature. Positive great average of the hydropathy value (GRAVY) scores mean hydrophobicity, as our result is 0.489.

The solubility analysis performed through Protein-Sol described a score of 0.382, which is lower than the tool threshold of 0.45. The threshold corresponds to *E. coli* solubility, and scores higher than 0.45 has a higher solubility average.

### Antigenicity, cytokine-inducing potential, and conformational B cell epitopes

The application of multi-epitope protein in cell-based in vitro platforms depends on its ability to be antigenic and to induce cytokine production. The designed protein had a predicted antigenicity score of 0.5596 through VaxiJen which means it is probably antigenic. The tool IFNepitope predicted two epitopes as probable IFN-γ inducer, and IL-4Pred and ProInflam predicted five and IL-10Pred four epitopes (Table 2). ElliPro predicted six linear and two conformational epitopes with scores greater than 0.7 (Table 3).

**Table 2:** Predicted IFN-γ inducer, IL-4Pred, and ProInflam-inducing epitopes

	Epitopes	Score
IFN	AILVTGFLWPAWLVT	0.02
	TRFVAAHGAYLVWLE	0.29
ProInflam	AVLWELGYRRFAYVD	0.95
	GVTYHYIDVPARTFA	0.84
	WGNWAKIFFNNKGVV	0.94
	YVHASLLAQNNTRVW	0.87
	TRFVAAHGAYLVWLE	0.70
IL-4	LAIASPCAYFLVYEP	0.27
	AVLWELGYRRFAYVD	0.35
	GVTYHYIDVPARTFA	1.30
	WGNWAKIFFNNKGVV	1.28
	TRFVAAHGAYLVWLE	0.22
IL-10	AVLWELGYRRFAYVD	0.54
	YVHASLLAQNNTRVW	0.56
	AILVTGFLWPAWLVT	0.35
	VLVFDAHRGMVVGSP	0.32

**Table 3:** The B cell conformational epitopes with a PI score greater than 0.7

Residues	Score
A324, A325, A327, I328, L329, V330, T331, G332, F333, L334, A336, A337, Y338, V339, T340, G341, F342, L343, W344, P345, A346, W347, A348, A349, Y350, and T353	0.80
R235, A237, Y238, V239, A240, A241, Y242, V243, L244, W245, E246, L247, G248, Y249, R250, R251, A252, A253, Y254, E255, L256, G257, Y258, R259, R260, Y278, H279, G281, N282, W283, A284, K285, F287, A288, A289, Y290, R291, Y302, T371, A372, Y374, I375, M376, S377, T378, I379, F380, G381, Q382, V383, A384, A385, Y386, L387, V388, F389, D390, A391, H392, R393, G394, M395, A396, A397, M399, V400, V401, N403, I404, L406, V407, A408, Y410, F411, A413, A414, H415, G416, A417, Y418, L419, A420, A421, Y422, K423, P424, E425, I426, L427, T428, R429, and L431	0.73

### In silico cloning

**Fig. 3:**
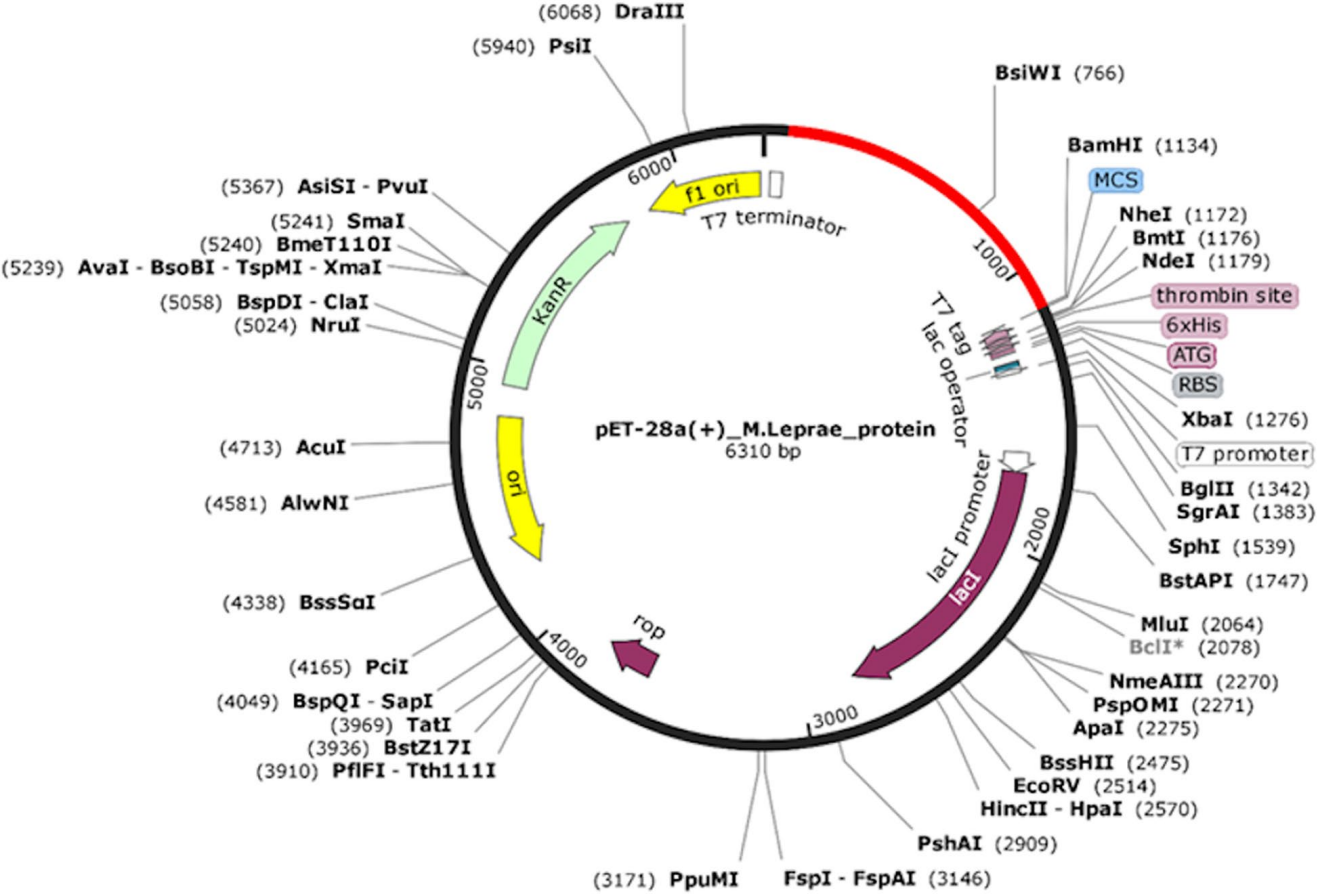
In silico cloning. The recombinant multi-epitope DNA sequence cloned into the pET28a( +) (Addgene) expression vector, represented in red color. The insert was added between the *BipI* and *BamHI* restriction sites

The codon adaptation Jcat software analysis showed that the GC content of the constructed sequence is 56.07%, and the CAI (Codon adaptation index) index is 1.0. Both are within the parameter range, which is important to measure the cloning and expression potential. In order to construct the cloning vector, through the SnapGene tool, restriction site sequences of the enzymes *BipI* and *BamHI* were inserted in the expression vector pET28a( +) (Addgene) totalizing 6310 base pairs in the complete clone length (Fig. 3).

## Discussion

In fact, there is a successful treatment for leprosy. However, the damage caused by the disease, due to the lack of accurate and early diagnosis, causes irreversible damage which highlights the need for highly sensitive detection tools for rapid diagnosis and epidemiological surveillance of the disease [35]. Also, in addition to being a neglected disease, many studies consider only *M. leprae* as the focus of their studies. Here, we chose to develop a universal chimeric protein that can identify not only *M. leprae*, but also *M. lepromatosis* infection, increasing the chance of diagnosis.

For being fastidious bacteria, *M. leprae* and *M. lepromatosis* were never cultivated in axenic media [8], which heightens the difficulty to work with them. That said, bioinformatics is an optimistic alternative, as it enables us to run innumerous tests without the bacteria, only with its genome.

ML0091, ML2380, and ML2346 were first described by Cole et al. in 2001 [69]. The first ones are similar to *M. tuberculosis* Rv3810 and Rv0455c proteins, but, as it was described, no homology was found in our construct. On the other hand, ML2346 has no known homology [13, 59]. As to its function, ML0091 is a 28-kDa antigen precursor, ML2380 is a possible secreted protein, and ML2346 is a hypothetical protein [69].

Duthie et al. tested ML0091 and ML2346 against the patients’ sera from Goiânia, Brazil, having a positive response in 71% and 29% of the cases, respectively [14]. The protein ML2328 was also used in the construction of LepVax (a subunit vaccine against *M. leprae*) by Duthie et al. [4].

The other proteins used were predicted by our group [37] in a reverse vaccinology approach; NP_301958.1, NP_302056.1, NP_302292.1, and NP_302503.1 are secreted proteins, and NP_302185.1, NP_301196.1, and NP_302342.1 are putative surface-exposed proteins. All these proteins are components of the core genome from four strains of *M. leprae* and two strains of *M. lepromatosis*. Since the definition of the core genome is to be present in all strains analyzed, it makes our recombinant multi-epitope protein a good candidate to diagnose the disease caused by any of them.

Given that diagnosis of leprosy is essential for treatment initiation and the earlier it begins, the better the response [54], many are the attempts to create a diagnostic approach that can detect leprosy in all its spectrum [63] and before any clinical sign [54, 63, 64], since infected individuals can spread the disease even before that [16]. Even though PCR is effective [63], leprosy is endemic in areas of difficult access or poverty, which makes its laboratory diagnosis hard [65]. Here, we propose a chimeric protein that has the potential to detect both humoral and cellular responses, which is established as efficient in diagnosing leprosy [10, 66, 67, 70] even in a not-so-controlled environment and with a lower cost than PCR.

Immunoinformatics is an in silico approach that helps in predicting epitopes that have a greater chance to be immunogenic; nevertheless, it may not be accurate if we take into consideration that proteins undergo unique biological complex processes driven by genetics to be presented as an epitope by a cell [20, 22, 71]. Nonetheless, several multi-epitope constructs are already described as being effective, for example, LID-1, a fusion construct of ML0405 and ML2331 that can diagnose MB leprosy 6 to 8 months prior to the onset of clinical symptoms [14]. LepReact, a delayed-type hypersensitivity skin test, made from LID-1, was able to detect antigen-specific immune responses from *M. leprae* in guinea pigs and armadillos [72]. As to other diseases, Chagas disease detection can be improved using TcF43 and TcF26, proteins derived from the fusion of selected *T. cruzi* TR proteins [28]; Yin et al. validated a high-accuracy ELISA assay using a recombinant protein for diagnosis of human brucellosis [27]; Ebrahimi et al. developed a multi-epitope protein with potential epitopes for the diagnosis of human toxocariasis [32].

Most of these tests are directed to humoral immunity, which is one of our goals, since it is less expensive and has a good accuracy in LL and MB leprosy, where antibodies are more present [8–10]; however, cellular assays may enable the detection of TT and PB leprosy. Sampaio et al. described IFN-γ secretion upon antigen-specific stimulation as an indicator of progression to the tuberculoid pole and IL-4 or IL-5 as an indicator of progression to the lepromatous pole [73].

T cell interferon-gamma release assays (IGRA) were developed as an alternative for delayed-typed hypersensitivity tests for latent tuberculosis diagnosis, reducing false-positive results [74, 75]. Since we found two IFN-γ, five IL-4, four IL-10 inducing epitopes, and five epitopes that induce pro-inflammatory responses within our protein, these properties point to a recombinant multi-epitope protein that can be used in a cytokine production assay, similar to the aforementioned IGRA, being able to detect different cellular immune profiles associated to different clinical manifestations of leprosy. IL-10 together with IL-4 is known to be associated with LL pole and MB leprosy while IFN-γ associated with other proinflammatory cytokines and characterized the TT pole and PB leprosy [73, 76–78].

With results in both the humoral and cellular response, this protein will be able to diagnose leprosy without much difficulty due to the detection of both ends of its spectrum. Other studies using immunoinformatics to construct multi-epitope proteins for diagnosis purposes had good results in silico, but they lacked sensitivity and specificity in ex vivo [79] or had strong specificity and weak sensibility [80].

## Conclusion

This novel recombinant multi-epitope protein has a huge immunological potential, is stable, and can be lyophilized to be used in ELISA plates or even in biosensors, which are user-friendly diagnosis tools, facilitating translation into human sample tests.

## Supplementary Information

**Additional file 1: Table S1.** List of Mycobacterium leprae and M. lepromatosis proteins selected for the analysis with their antigenicity value predicted through VaxiJen. **Table S2.** Number of B cell epitopes. **Table S3.** The total numbers of MHC I epitopes. **Table S4.** The total numbers of MHC II epitopes. **Table S5.** Number of epitopes in MHC II and I filtering. **Table S6.** The Immunogenicity Scores of MHC I predicted epitopes. **Fig. S1.** PSIPRED secondary structure prediction.

## Data Availability

All the required methods and results to reproduce this article are provided as part of the paper and the supplementary material.

## Abbreviations

ANN: Artificial neural network
BT: Borderline tuberculoid
BB: Borderline-borderline
BL: Borderline lepromatous
CAI: Codon Adaptation Index
CTL: Cytotoxic T lymphocytes
ELISA: Enzyme-linked immunosorbent assay
IL: Interleukin
IFN-γ: Interferon gamma
IC50: Half maximal inhibitory concentration
IEDB: Immune Epitope Database
IGRA: Interferon-gamma release assays
LL: Lepromatous leprosy
MDT: Multidrug therapy
MHC: Major histocompatibility complex
MTT: Multiple-template threading
MB: Multibacillary
nM: Nanomolar
PB: Paucibacillary
PCR: Polymerase chain reaction
PSSM: Position-specific scoring matrix
SMM: Scoring matrix method
TL: Tuberculoid leprosy
Th: T helper cells
WHO: World Health Organization
