# Supplementary material for: In silico designing of a recombinant multi-epitope antigen for leprosy diagnosis
Source: J Genet Eng Biotechnol. 2022 Sep 2;20:128. doi: 10.1186/s43141-022-00411-7 (PMC9440174; doi:10.1186/s43141-022-00411-7)
Supplement: Supplementary file 1 — Additional file 1: Table S1. List of Mycobacterium leprae and M. lepromatosis proteins selected for the analysis with their antigenicity value predicted through VaxiJen. Table S2. Number of B cell epitopes. Table S3. The total numbers of MHC I epitopes. Table S4. The total numbers of MHC II epitopes. Table S5. Number of epitopes in MHC II and I filtering. Table S6. The Immunogenicity Scores of MHC I predicted epitopes. Fig. S1. PSIPRED secondary structure prediction. [file 43141_2022_411_MOESM1_ESM.docx]

| **Protein** | **Name** | **Overall Prediction for the Protective Antigen** |
| --- | --- | --- |
| **ML0091** | 28 KDa antigen precursor | 0.5993 |
| **ML0405** | conserved hypothetical protein | 0.5507 |
| **ML1556** | initiation factor IF-2 | 0.7560 |
| **ML1633** | possible secreted hydrolase | 0.4079 |
| **ML2028** | antigen 85A, mycolyltransferase | 0.5759 |
| **ML2055** | probable cell surface protein | 0.4885 |
| **ML2331** | possible secreted protein | 0.7658 |
| **ML2346** | L-lactate dehydrogenase | 0.4681 |
| **ML2380** | possible secreted protein | 0.3004 |
| **ML2531** | possible cell surface protein | 0.5242 |
| **NP_301196.1** | Antigen 85-C precursor 85C] Antigen 85 complex C]  Ag85C]  Mycolyl transferase 85C]  EC 2.3.1.-] | 0.4657 |
| **NP_301663.1** | Putative membrane protein MmpS3 | 0.6988 |
| **NP_301805.1** | Putative lipoprotein IprE Precursor | 0.3909 |
| **NP_301958.1** | Possible membrane protein | 0.3909 |
| **NP_302056.1** | Hypothetical protein | 0.4972 |
| **NP_302185.1** | Hypothetical protein | 0.4572 |
| **NP_302232.1** | Invasion protein – Putative exported p60 protein homologue | 0.5827 |
| **NP_302292.1** | Hypothetical protein | 0.5468 |
| **NP_302342.1** | PPE Family protein | 0.6320 |
| **NP_302490.1** | Protease | 0.6261 |
| **NP_302503.1** | Multimodular transpeptidasetransglycosylase  EC 2.4.1.129] | 0.5410 |

**Table A1** - List of Mycobacterium leprae and M. lepromatosis proteins selected for the analysis with their antigenicity value predicted through VaxiJen

| **B cell epitopes** | | | |
| --- | --- | --- | --- |
| **Protein** | **ABCpred** | **Lbtope** | **Overlapping** |
| **ML0091** | 22 | 70 | 6 |
| **ML0405** | 39 | 106 | 10 |
| **ML1556** | 98 | 253 | 25 |
| **ML1633** | 51 | 178 | 17 |
| **ML2028** | 32 | 82 | 16 |
| **ML2055** | 30 | 88 | 11 |
| **ML2331** | 23 | 54 | 4 |
| **ML2346** | 30 | 94 | 11 |
| **ML2380** | 14 | 46 | 6 |
| **ML2531** | 10 | 17 | 2 |
| **NP_301196.1** | 31 | 66 | 9 |
| **NP_301663.1** | 28 | 93 | 9 |
| **NP_301805.1** | 20 | 52 | 6 |
| **NP_301958.1** | 31 | 110 | 15 |
| **NP_302056.1** | 36 | 95 | 8 |
| **NP_302185.1** | 37 | 140 | 13 |
| **NP_302232.1** | 25 | 51 | 6 |
| **NP_302292.1** | 25 | 91 | 9 |
| **NP_302342.1** | 44 | 103 | 13 |
| **NP_302490.1** | 22 | 29 | 2 |
| **NP_302503.1** | 81 | 280 | 29 |
| **Total** | **729** | **2098** | **227** |

**Table A2 – Number of B cell epitopes**

| **MHC I** | | | |
| --- | --- | --- | --- |
| **Protein** | **IEDB** | **NET CTL** | **Overlapping** |
| **ML0091** | 48 | 33 | 29 |
| **ML0405** | 114 | 63 | 56 |
| **ML1556** | 235 | 140 | 122 |
| **ML1633** | 136 | 78 | 69 |
| **ML2028** | 83 | 60 | 49 |
| **ML2055** | 69 | 42 | 36 |
| **ML2331** | 56 | 37 | 30 |
| **ML2346** | 95 | 57 | 48 |
| **ML2380** | 51 | 31 | 29 |
| **ML2531** | 20 | 13 | 10 |
| **NP_301196.1** | 293 | 48 | 48 |
| **NP_301663.1** | 285 | 52 | 52 |
| **NP_301805.1** | 49 | 28 | 22 |
| **NP_301958.1** | 57 | 33 | 29 |
| **NP_302056.1** | 83 | 53 | 47 |
| **NP_302185.1** | 94 | 56 | 46 |
| **NP_302232.1** | 55 | 33 | 27 |
| **NP_302292.1** | 60 | 37 | 29 |
| **NP_302342.1** | 141 | 97 | 84 |
| **NP_302490.1** | 52 | 37 | 29 |
| **NP_302503.1** | 197 | 118 | 101 |
| **Total** | 2273 | 1146 | 992 |

**Table A3 - The total numbers of MHC I epitopes**

| **MHC II** | | | |
| --- | --- | --- | --- |
| **Protein** | **IEDB** | **NET 2.3** | **Overlapping** |
| **ML0091** | 23 | 65 | 21 |
| **ML0405** | 74 | 155 | 56 |
| **ML1556** | 67 | 303 | 65 |
| **ML1633** | 28 | 222 | 28 |
| **ML2028** | 64 | 154 | 57 |
| **ML2055** | 7 | 273 | 7 |
| **ML2331** | 31 | 92 | 31 |
| **ML2346** | 61 | 145 | 50 |
| **ML2380** | 33 | 58 | 25 |
| **ML2531** | 6 | 40 | 4 |
| **NP_301196.1** | 5 | 211 | 5 |
| **NP_301663.1** | 14 | 127 | 14 |
| **NP_301805.1** | 12 | 108 | 12 |
| **NP_301958.1** | 2 | 63 | 2 |
| **NP_302056.1** | 10 | 207 | 10 |
| **NP_302185.1** | 29 | 240 | 28 |
| **NP_302232.1** | 3 | 145 | 3 |
| **NP_302292.1** | 13 | 120 | 13 |
| **NP_302342.1** | 90 | 352 | 90 |
| **NP_302490.1** | 18 | 143 | 18 |
| **NP_302503.1** | 47 | 511 | 47 |
| **Total** | 637 | 3734 | 586 |

**Table A4 - The total numbers of MHC II epitopes**

|  | **MHC II FILTERING** | **MHC I FILTERING** | |
| --- | --- | --- | --- |
| **Protein** | **B cell epitopes overlapping** | **Immunogenicity** | **Overlapping of MHC II with overlapping in B** |
| **ML0091** | 1 | 7 | 1 |
| **ML0405** | 4 | 27 | 0 |
| **ML1556** | 1 | 49 | 0 |
| **ML1633** | 2 | 21 | 0 |
| **ML2028** | 5 | 15 | 0 |
| **ML2055** | 1 | 9 | 0 |
| **ML2331** | 2 | 9 | 0 |
| **ML2346** | 8 | 21 | 5 |
| **ML2380** | 3 | 15 | 1 |
| **ML2531** | 0 | 1 | 0 |
| **NP_301196.1** | 1 | 21 | 1 |
| **NP_301663.1** | 1 | 0 | 0 |
| **NP_301805.1** | 0 | 11 | 0 |
| **NP_301958.1** | 1 | 11 | 6 |
| **NP_302056.1** | 1 | 15 | 1 |
| **NP_302185.1** | 1 | 16 | 1 |
| **NP_302232.1** | 0 | 11 | 0 |
| **NP_302292.1** | 1 | 11 | 1 |
| **NP_302342.1** | 5 | 39 | 1 |
| **NP_302490.1** | 0 | 10 | 0 |
| **NP_302503.1** | 2 | 31 | 2 |
| **Total** | 40 | 350 | 20 |

**Table A5 – Number of epitopes in MHC II and I filtering**

| **Protein** | **Immunogenicity** | |
| --- | --- | --- |
|  | **Epítopo** | **Score** |
| **ML0091** | SPCAYFLVY | 0,14323 |
| **ML2346** | LGYRRFAYV | 0,24234 |
|  | VLWELGYRR | 0,22087 |
|  | ELGYRRFAY | 0,22806 |
|  | YIDVPARTF | 0,14113 |
| **ML2380** | HWGNWAKIF | 0,15278 |
| **NP_301196.1** | RWKWHDPYV | 0,19375 |
| **NP_301958.1** | VLIFAAILV | 0,34177 |
|  | VTGFLWPAW | 0,34001 |
|  | LVTGFLWPA | 0,33072 |
|  | FLWPAWLVT | 0,32211 |
|  | FAAILVTGF | 0,22724 |
|  | AILVTGFLW | 0,19996 |
| **NP_302056.1** | IMSTIFGQV | 0,18608 |
| **NP_302185.1** | LVFDAHRGM | 0,19235 |
| **NP_302292.1** | MVVTNIGLV | 0,19356 |
| **NP_302342.1** | FVAAHGAYL | 0,14633 |
| **NP_302503.1** | KPEILTRYL | 0,23368 |

**Table A6 – The Immunogenicity Scores of MHC I predicted epitopes**


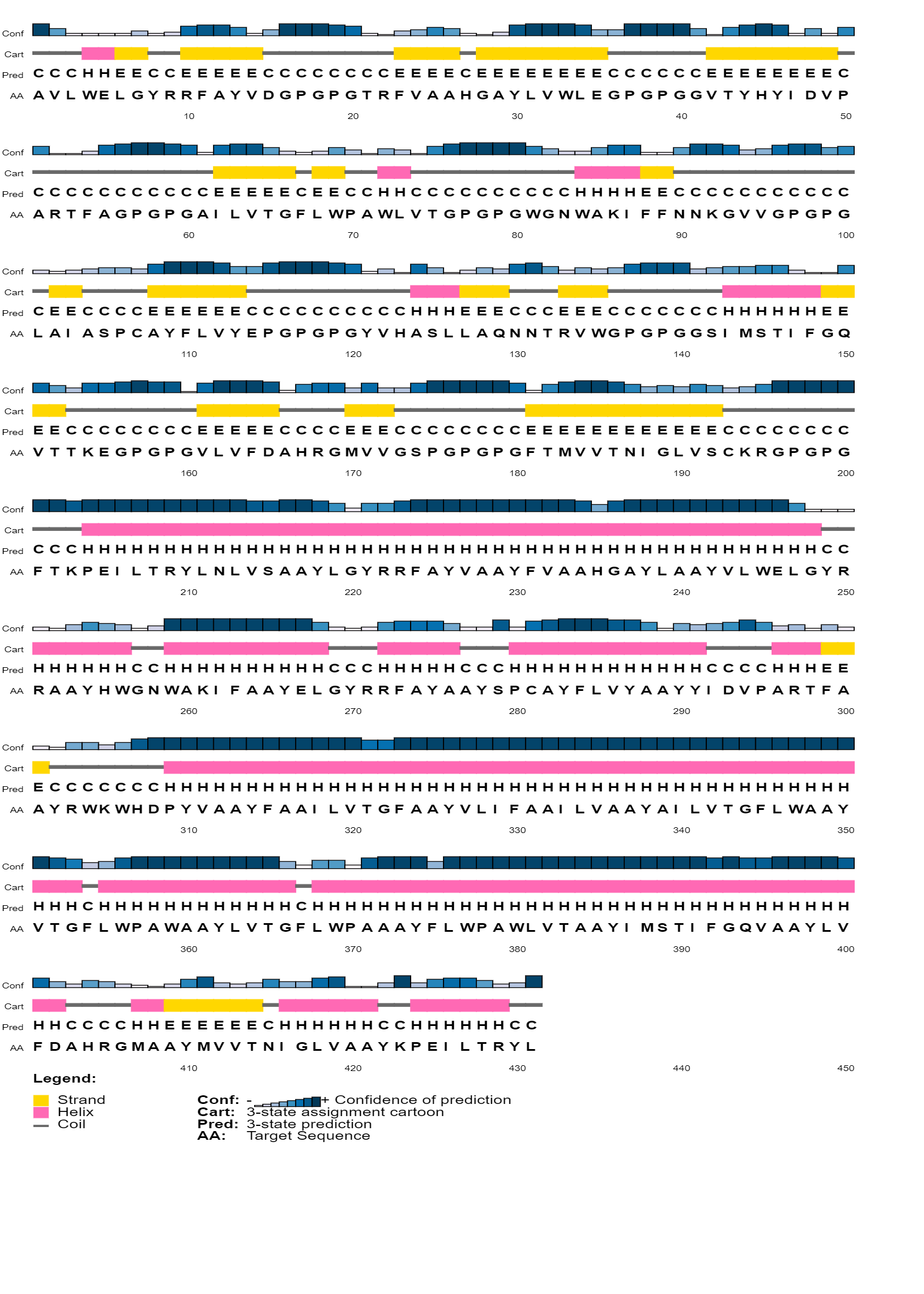


**Figure A1 - PSIPRED secondary structure prediction**
